# Recommendations for Risk Categorization and Prophylaxis of Invasive Fungal Diseases in Hematological Malignancies: A Critical Review of Evidence and Expert Opinion (TEO-4)

**DOI:** 10.4274/tjh.2014.0277

**Published:** 2015-05-08

**Authors:** Can Boğa, Zahit Bolaman, Seçkin Çağırgan, İhsan Karadoğan, Mehmet Ali Özcan, Fahir Özkalemkaş, Rabin Saba, Mehmet Sönmez, Esin Şenol, Hamdi Akan, Murat Akova

**Affiliations:** 1 Başkent University Faculty of Medicine Hospital, Department of Hematology, Adana, Turkey; 2 Adnan Menderes University Faculty of Medicine, Division of Hematology, Aydın, Turkey; 3 Medical Park İzmir Hospital, Clinic of Hematology and Bone Marrow Transplantation Center, İzmir, Turkey; 4 Medstar Antalya Hospital, Clinic of Hematology and Stem Cell Transplantation, Antalya, Turkey; 5 Dokuz Eylül University Faculty of Medicine, Department of Hematology, İzmir, Turkey; 6 Uludağ University Faculty of Medicine, Department of Hematology, Bursa, Turkey; 7 Medstar Antalya Hospital, Clinic of Infectious Diseases, Antalya, Turkey; 8 Karadeniz Technical University Faculty of Medicine, Department of Hematology, Trabzon, Turkey; 9 Gazi University Faculty of Medicine, Department of Infectious Diseases, Ankara, Turkey; 10 Ankara University Faculty of Medicine, Department of Hematology, Ankara, Turkey; 11 Hacettepe University Faculty of Medicine, Department of Infectious Diseases, Ankara, Turkey

**Keywords:** Hematological malignancy, Invasive fungal infections, Prophylaxis, Risk

## Abstract

This is the last of a series of articles on invasive fungal infections prepared by opinion leaders in Turkey. The aim of these articles is to guide clinicians in managing invasive fungal diseases in hematological malignancies and stem cell transplantation based on the available best evidence in this field. The previous articles summarized the diagnosis and treatment of invasive fungal disease and this article aims to explain the risk categorization and guide the antifungal prophylaxis in invasive fungal disease.

## Abstract (tr)

Bu makale Türkiye’de invazif fungal enfeksiyon ile uğraşan uzmanlar tarafından hazırlanan bir seri yazının sonuncusudur. Bu makaleler hematolojik malignitelerde ve kök hücre nakli hastalarında invazif fungal hastalıkların yönetimini eldeki kanıtların ışığında en iyi hale getirmeyi amaçlamaktadır. İlk yazılar tanı ve tedaviyi özetlerken, bu makale invazif fungal hastalıkta risk kategorizasyonu ve profilaksiyi ele almaktadır.

## INTRODUCTION

### Dr. Murat Akova 

Invasive fungal diseases (IFDs) continue to be an important cause of morbidity and mortality during the course of disease in patients with hematological malignancies and/or stem cell transplantation [1]. The lack of a cheap, easily applied diagnostic test with high sensitivity and specificity, as well as the serious mortality caused by the delay in diagnosis, has brought prevention of fungal infections to the forefront. While recently developed effective new antifungal medications with a wide spectrum of activity make prophylactic application attractive in high-risk patient groups, the wide use of serological and molecular biological diagnostic tools, like high-resolution computerized tomography examinations, serum galactomannan and beta-D-glucan tests, and PCR applications provides effective treatment options for selected patients in the early period [2]. The most important concerns about prophylaxis are additional costs, unwanted side effects due to antifungal use, and development of resistance due to the use of unnecessary antifungal antibiotics in many patients in order to prevent fungal infection-related mortality in one patient. Therefore, before routine antifungal prophylaxis (AFP) is given to high-risk patient groups, the above-mentioned risk-benefit relationship should be necessarily reviewed. If an example is to be given, in order to decrease infection incidence by 50%, the number of patients who should receive prophylaxis is 100 in an environment where fungal infection prevalence is 2%, and this figure drops to 44 in the event that the prevalence is 4.5% [3]. Another point that should be kept in mind before application of prophylaxis is that although a decrease in fungal infection incidence and fungal-related mortality has been provided by AFP in numerous clinical studies performed to date, only 2 studies have been able to show a decrease in the general mortality of high-risk patients [4]. All these findings prove that selective action should be taken in high-risk patients regarding AFP.

## Determination of Risk and Targets of Prophylaxis

### Dr. Fahir Özkalemkaş

Patients with hematological malignancies and allogeneic stem cell transplantation are the primary risk groups for IFD [5,6]. It has been known for a long time that early treatment initiation in patients with IFD is one of the major determinants of successful treatment and decreased mortality [7,8,9]. Despite all the recent advances in diagnostic testing, the absence of rapid and reliable diagnostic tests in IFD diagnosis enhances the importance of risk determination. When the prevalence and mortality rates are taken into consideration, well-defined high-risk patients constitute the group that will benefit from the prophylactic use of an effective antifungal agent. Therefore, determination of the correct risk level forms the basis of AFP. Furthermore, besides an increase in mortality, IFDs lead to delays in scheduled treatments (chemotherapy, stem cell transplantation), longer hospital stays, and increased treatment costs [10,11,12,13].

Invasive aspergillosis and invasive candidiasis are the most important entities of IFD. A third important group is invasive mucormycosis infection, with a relatively low prevalence but a high mortality rate [5,6]. Apart from common risk factors such as neutropenia and long-term myelosuppression, risk profile varies depending on the causative agent. For example, while the presence of numerous spores in inspired air and factors facilitating the passage of these spores through respiratory mucous membranes and reduced phagocytosis in the tissues play the major role in aspergillosis infections, diminished general phagocytic activity, dense colonization in the gastrointestinal tract mucosa, and mucosal damage due to chemo-/radiotherapy come to the forefront in invasive candidiasis. For mucormycosis, in addition to defective phagocytosis, other factors such as prior steroid use and the presence of metabolic acidosis should also be taken into consideration [1,5,14,15].

Hematological malignancies are widely heterogeneous in terms of risk. Among patients with hematological malignancies, acute leukemia patients, particularly those with acute myeloid leukemia (AML) receiving remission induction chemotherapy, are at significantly higher risk [6,13]. High-risk myelodysplastic syndrome (MDS) is treated similarly to AML, as both have similar biological behavior; hence, MDS patients receiving remission induction chemotherapy are accepted to have the same risk profile as AML patients [16]. Likewise, hematopoietic stem cell transplantation patients are also heterogeneous. Risk is especially lower in patients with autologous transplantation than in those with allogeneic transplantation. Among allogeneic stem cell recipients, risk is significantly increased in those with graft-versus-host disease (GVHD) and long-term steroid use [17]. Another point that should not be forgotten is that risk profile may change over time due to the use of new treatment agents. For instance, the use of new monoclonal antibodies may alter the risk profile of chronic lymphocytic leukemia patients who are considered to be at low risk [18]. Similarly, use of new protocols in both the preparative regimen and GVHD prophylaxis in allogeneic stem cell transplantation, different stem cell sources (bone marrow, peripheral blood, umbilical cord blood), donor type (unrelated donors, relatives with perfect or partial match), stem cell manipulation (T-cell reduction), and superimposed infections (cytomegalovirus, respiratory syncytial virus infections) may lead to remarkable changes in the risk profile [10,17,19,20,21,22,23,24,25].

Apart from the comorbid conditions, the most important parameter in IFD development is neutropenia [26]. Both the depth and duration of neutropenia are important; more recently, an index taking these 2 parameters into account was reported to be important in predicting invasive mold infections [27]. In allogeneic transplants, in addition to neutropenia, development of long-term lymphopenia particularly enhances the risk of invasive aspergillosis [17]. 

Although specific polymorphisms (toll-like receptor 4, plasminogen alleles, dectin-1, TNF-1A) in some genes affecting natural immunity have been reported in recent years to make significant changes in invasive aspergillosis risk, these parameters are far from practical for use in the determination of treatment approach [28,29,30,31,32,33]. A recent study demonstrated that genetic deficiency of pentraxin 3 (PTX3) affects the antifungal capacity of neutrophils and may contribute to the risk of invasive aspergillosis in patients treated with hematopoietic stem cell transplantation [34].

Finally, in daily practice, particularly in Turkey, it is wise to underline the importance of environmental factors such as ‘air quality’ and personal factors like ‘colonization’ and ‘prevention’ in risk determination. Causative molds in the air may be at different concentrations in different geographic regions and may show seasonal variations in the same region [1,20,35]. In this respect, positive-pressure HEPA filters can be of critical importance in reducing the risk in risky regions and periods. Colonization during hospitalization is considered among the risk factors [36]. Other environmental control measures, hand-washing being the leading one, may reduce the risk [37].

## Timing of Prophylaxis

### Dr. Esin Şenol

There is no standard approach or recommendation regarding the optimal timing of prophylaxis in the guidelines and prophylaxis protocols. It is understood from prophylaxis studies that prophylaxis initiation times are different: at the time of hospitalization, or at the beginning or at the end of chemotherapy. In 2 important studies on primary prophylaxis using posaconazole, AML or MDS patients receiving chemotherapy had prophylaxis initiated together with chemotherapy in those not using anthracycline or 24 h after anthracycline in those using anthracycline. It was planned to be continued until resolution of neutropenia (ANC>500/mm3), fungal infection development, or for 12 weeks [mean of 23 days (1-110 days, 29±21)]. In patients with allogeneic stem cell transplantation and GVHD, it was planned to be given at the time of development of acute GVHD of 2-4 degree or chronic disseminated GVHD at a dose of 1 mg/kg/day for acute GVHD and 0.8 mg/kg every other day for chronic GVHD, or at the beginning of 2 or more immunosuppressive agents without steroids, and scheduled to be continued for 112 days [38,39]. The reason for starting prophylaxis along with chemotherapy is that the oral antifungals used for prophylaxis reach their plasma saturation levels in the neutropenic period when fungal infection risk is the highest. This period is 5 days if voriconazole is used orally and 7-10 days if posaconazole is used [2,40,41]. However, there is concern that the azole antifungals mostly used for prophylaxis may interact with drugs used in chemotherapy regimens.

Fluconazole prophylaxis (not involving molds) is given to allogeneic stem cell transplantation recipients before engraftment, in the beginning or immediately after the preparative regimen. There is a strong recommendation for giving prophylaxis for at least 3-6 months after engraftment; however, this period can be prolonged if treatment-related immunosuppression is caused by drugs such as corticosteroids [42]. 

The single remarkable end-point for the timing of prophylaxis termination for autologous stem cell transplantation recipients and for patients receiving AML/MDS chemotherapy is the resolution of neutropenia. Additionally, prophylaxis is discontinued in the event of conditions like drug intolerance, development of a new fungal infection, and drug-related side effects. However, there are still uncertainties in the timing of termination as well as the timing of initiation of prophylaxis, especially in allogeneic stem cell transplantation recipients [43].

## Agents Used in Prophylaxis

### Dr. Mehmet Sönmez

Polyene, azole, and echinocandin-class antifungal agents, including fluconazole, itraconazole, posaconazole, voriconazole, micafungin, anidulafungin, caspofungin, amphotericin B deoxycholate, and liposomal amphotericin B, have been used for AFP [38,39,44,45,46,47,48,49,50,51,52]. Toxicity, drug interactions, costs, effects of the used antifungal agent on fungal diagnostic tests, and risk of developing resistant fungal infections should be taken into account in patients receiving antifungal prophylaxis. While a meta-analysis of the studies comparing the efficacy of antifungal prophylaxis in patients receiving AML/MDS induction treatment or in those undergoing allogeneic hematopoietic stem cell transplantation showed that antifungal prophylaxis decreased IFD prevalence and IFD-related mortality, similar effects were not observed in patients undergoing autologous stem cell transplantation. The incidence of IFD, and especially the incidence of Aspergillus infections, was found to be lower in patients receiving prophylaxis for molds. However, side effect-related discontinuation of the drug was found to be higher compared to the fluconazole-treated group. It was noted that that overall mortality was not changed [11,51,53]. Currently, echinocandins, apart from micafungin and anidulafungin, and polyene-group antifungals are not considered to be preferable prophylactic agents in spite of their wide antifungal activity spectrum because of intravenous use, side effect profiles, costs, and absence of sufficient data on prophylactic use. Therefore, azole-group drugs are generally preferred in antifungal prophylaxis. Although itraconazole, included in this group of drugs, is an effective agent, high discontinuation rates due to gastric intolerance, drug interactions, and variable bioavailability restrict its use. Fluconazole, with the lowest rate of side effects and drug interactions and an activity spectrum limited to Candida species, is recommended in patients undergoing allogeneic hematopoietic stem cell transplantation during neutropenia, but necessitates mold testing during prophylaxis. The demonstration that posaconazole is more effective in preventing IFD development in comparison to fluconazole led to the preferential use of posaconazole prophylaxis in AML/MDS patients receiving induction treatment and in those who developed GVHD after allogeneic hematopoietic stem cell transplantation. However, the necessity of taking posaconazole on a full stomach along with food rich in fats, drug interactions, and the necessity of drug level monitoring are among the factors that restrict its usage. Likewise, while voriconazole, an azole-group drug that should be used in therapeutic levels, is recommended to be used in patients undergoing allogeneic stem cell transplantation, it requires careful monitoring of hepatotoxicity, neurotoxicity, and drug interactions [2,54,55,56,57,58,59,60]. Furthermore, voriconazole is recommended to be used for secondary prophylaxis in patients with prior Aspergillus infection who require retreatment or allogeneic hematopoietic stem cell transplantation. On the other hand, in hospitals with a mold incidence of <5% and with HEPA filtration, monitoring of patients with diagnostic tests without antifungal prophylaxis for molds may also be a suitable approach [60,61,62]. 

## Monitoring of Prophylaxis

### Dr. Rabin Saba

After a decision is made about AFP and the drug that will be used, the efficacy and side effects of the chosen drug should be monitored. Primarily, the interaction of the drug with food and other drugs should be evaluated. The bioavailability of voriconazole increases when taken on an empty stomach, itraconazole capsule with food, and posaconazole with fatty food. The bioavailability of proton pump inhibitors or H2 receptor blockers decreases when used together with posaconazole or itraconazole [2,63]. Considering drug interactions, special care should be taken when using triazoles, which can be both the substrate and the inhibitor of cytochrome P (CYP) 450 isoenzymes. Each drug should be considered individually. For instance, posaconazole is metabolized by glucuronidation, not by the CYP system; however, it is a weak inhibitor of CYP 3A4. For this reason, if taken together with drugs inducing CYP enzymes, the serum concentration of triazoles other than posaconazole decreases. If taken together with triazoles, the serum concentration of drugs metabolized by CYP enzymes increases. It is contraindicated to use sirolimus with voriconazole and posaconazole [64]. When interactions with chemotherapeutic agents are considered, the best known example is the interaction between itraconazole and vincristine [65]. The increased neurotoxicity (by crossing the blood brain barrier) and the organ toxicity of vincristine is noteworthy. Antifungal drugs should also be monitored in terms of side effects [66]. While triazoles are particularly monitored regarding tolerability and hepatotoxicity, amphotericin B should be monitored in terms of infusion-related side effects, nephrotoxicity, and hypokalemia. The point that should not be forgotten is that prophylaxis should be used in conditions where the protective effects of prophylaxis are superior to the expected side effects. Discontinuation of the drug and/or switching to another drug should be considered in the case of side effects. 

## Drug Level Monitoring

While therapeutic drug monitoring generally gains importance for mold-active triazoles (itraconazole, voriconazole, and posaconazole), it is not recommended for echinocandin and polyene-group antifungals [2,63]. Measurement of serum concentrations is especially recommended in pharmacokinetically unstable patients (children, neonates, critical patients, those with organ dysfunction, etc.), in the suspicion of incompatibility, in the presence of drug interactions, when switching from the intravenous form of the drug to the oral form, and in patients with absorption problems such as diarrhea or GVHD. 

## Itraconazole

The bioavailability of itraconazole is variable and shows changes depending on the formulation. Bioavailability of the oral capsules increases with food and gastric acidity. The oral solution, which has better bioavailability, is much better absorbed when taken on an empty stomach and is not affected by gastric acidity. As the rates of breakthrough infections and mortality were found to be significantly higher at lower drug levels, it is required to maintain the serum concentrations at >8 mg/L (measuring both itraconazole and hydroxy-itraconazole levels) as measured by bioassay method, at <0.5-1 mg/L by high-performance liquid chromatography and mass spectrometry, and at <17 mg/L by bioassay to minimize gastrointestinal, neurological, and hepatic toxicity [67,68]. As the drug concentration will achieve a steady state within 2 weeks, the measurements should begin after 7 days [2,63,64,65,66,67,68]. 

## Voriconazole

As voriconazole shows nonlinear pharmacokinetics, changes in the dosage are not similarly reflected. Although the efficacy of prophylaxis monitoring has not been clearly demonstrated, serum concentrations are to be maintained at 1-5 mg/L with regard to toxicity [63,69,70]. It is recommended that serum concentrations should be measured within and after 5 days of use.

## Posaconazole

Due to its long half-life (34 h), and because the drug concentration achieves a steady state within 7 days, the first measurement is recommended to be performed after 1 week of use [63]. The plateau concentration is recommended to be >0.7 mg/L for efficient prophylaxis [63,71,72]. It was shown that the alveolar intracellular posaconazole level was 40-50 times greater than outside the cell and this might explain the efficacy of posaconazole prophylaxis in patients with low serum posaconazole levels [73]. As the alveolar tissue concentration is important for posaconazole, it has been stated that alveolar concentration rather than serum concentration will be required to be measured in the future [63].

In the follow-up of patients receiving mold prophylaxis for fungal infections, special care should be given to diagnostic test interpretation. Notably, the sensitivity of the galactomannan test is decreased in patients receiving posaconazole and voriconazole prophylaxis [74]. A study evaluating the place of Aspergillus polymerase chain reaction and galactomannan antigen in bronchoalveolar lavage fluid in diagnosis showed that anti-mold prophylaxis decreases sensitivity [75]. On the other hand, there are studies reporting that itraconazole prophylaxis had no effect on the molecular method used [76]. Therefore, sensitivity of molecular tests should also be interpreted with caution [77,78].

## Prophylaxis Failure

### Dr. Seçkin Çağırgan

AFP failure may be defined as the development of proven or probable IFD during prophylaxis, the requirement of empirical antifungal treatment, and the necessity of discontinuing the prophylaxis drug due to side effects or patient-related reasons [39].

AFP failure due to development of an IFD may be related to the activity spectrum of the prophylactic agent, development of infection with resistant fungal pathogens, and failure to provide effective blood levels of the drug. Although fluconazole prophylaxis significantly reduces Candida infections in patients with acute myelocytic leukemia and in those undergoing allogeneic stem cell transplantation, it is accompanied with an increase in the rates of invasive aspergillosis and other mold infections, as fluconazole is not active against molds [39,42,44,79]. Moreover, it has been shown that fluconazole prophylaxis increases colonization and development of infection with resistant non-albicans Candida species [80,81]. The risk of aspergillosis decreases if a broad-spectrum azole (itraconazole, voriconazole, or posaconazole) or an echinocandin effective against Aspergillus species is used; however, the probability of infections with other molds, especially Mucorales species, remains the same, as itraconazole, voriconazole, and echinocandins are not effective against Mucorales [82]. Failure in providing adequate serum levels is most frequently seen when using oral itraconazole and posaconazole and this may lead to prophylaxis failure and development of an IFD [2]. Particularly, patients with mucositis, nausea and vomiting, insufficient enteral intake, and diarrhea are at risk. 

Necessity of termination of prophylaxis or switching to another drug may be associated with patient intolerance or drug toxicity. Gastrointestinal intolerance and hepatotoxicity are the most common toxicity-related causes of AFP termination [2]. 

Prophylaxis using mold active agents, posaconazole being the leading one, has been demonstrated to significantly decrease the sensitivity of galactomannan testing [83,84]. Therefore, a preemptive AFP treatment approach based on galactomannan antigen monitoring will not be safe in these patients; hence, an empirical treatment approach is recommended.

A detailed diagnostic study should be started in patients with prophylaxis failure if symptoms and clinical findings indicative of an IFD are present. If possible, the pathogen should be detected (microscopic examination in suitable samples, culture, histopathological examination; bronchoscopy, galactomannan testing in bronchoalveolar lavage fluid in the presence of a pulmonary lesion, etc.) [2].

Which antifungal agent should be selected in AFP failure characterized by IFD development or the requirement of initiating empirical fungal treatment? As a general rule, a change in the antifungal agent class should be considered if an IFD is suspected [2]. The majority of cases with prophylaxis failure are associated with the development of pulmonary infiltrates. During oral mold-active azole prophylaxis, switching to liposomal amphotericin B should be considered if aspergillosis is suspected (galactomannan positivity) and effective serum levels of itraconazole or posaconazole can be achieved or if drug level monitoring is not available [2,82]. If low serum levels of itraconazole or posaconazole have been shown, intravenous voriconazole can be used [2]. If galactomannan antigen testing is negative or is not available and a mold-active azole or echinocandin effective against Aspergillus strains has been used in prophylaxis, the risk of Aspergillus infection decreases; however, the probability of infections with other filamentous fungi, particularly Mucorales, remains, as itraconazole, voriconazole, and echinocandins are not effective against Mucorales strains. Posaconazole is active against some Mucorales strains; however, effective serum concentrations usually cannot be achieved. In these conditions, liposomal amphotericin B, with the widest spectrum of activity (Candida, Aspergillus species, Cryptococcus, Fusarium, Mucorales, and endemic fungi), is the antifungal drug that should be chosen. 

Azoles should not be used empirically in the case of prior azole prophylaxis [58].

## Secondary Prophylaxis

### Dr. Mehmet Ali Özcan

During cytotoxic treatment, there is a substantial risk of recurrent invasive fungal infection in patients who “survived” the first invasive fungal infection. This rate is between 16% and 33% in the published series and IFD-related mortality reaches up to 88% [85,86,87,88,89]. Application of antifungal drugs for the management of this risk is called “secondary prophylaxis”. The most important problem in this area is that there is still no prospective randomized study on this subject. Therefore, evaluations are based on available experiences with different applications of antifungal agents. Amphotericin formulations, fluconazole, itraconazole, caspofungin, voriconazole, and posaconazole can be used in secondary prophylaxis depending on the use and success of these agents in primary treatment [90] (Table 1). While secondary prophylaxis seems to be effective according to the information obtained from case series and the few prospective secondary prophylaxis studies published to date, sufficient evidence to make a recommendation on the use of “which agent”, “what dose”, and “for how long” has not yet been provided. 

In manuscripts evaluating secondary prophylaxis, probable risk factors, mainly neutropenia duration, state of underlying disease, presence of GVHD, and steroid use, are found to be important.

The European Conference on Infections in Leukemia guidelines recommend secondary prophylaxis with an evidence level of AII, and, instead of recommending a certain agent, they recommend that secondary prophylaxis should be based on the causative agent of the prior invasive fungal infection and treatment success [58]. 

In the decision-making period of secondary prophylaxis, the selected patients with sequel lesions are to be evaluated in terms of receiving chemotherapy or surgical resection before transplantation. As this group of patients is small in number in case series, it would be suitable to evaluate this subgroup separately in clinical studies.

## Environmental Protection

### Dr. Can Boğa

The basic principle in AFP is to use drugs with proven efficacy and high evidence levels. This section of the article mainly discusses subjects associated with environmental factors. 

Nosocomial fungal infections are mainly transmitted by air, and less frequently by the oral route. It is well known that hospital construction and repair activities may increase the fungal spore concentrations in the air and that they are associated with the frequency of IFDs [96,97]. 

## Organizations That Recommend the Use of HEPA Filters in Critical Areas

Areas such as bone marrow transplantation units, in which immunosuppressed patients that require protection from infectious agents are monitored, and the isolation areas in which infected patients are monitored are defined as critical areas. The US Centers for Disease Control and Prevention (CDC) and the Spanish Society of Infectious Diseases and Clinical Microbiology recommend that these areas should be separated from other areas; heating, water systems, and ventilation conditions should be specially organized; and HEPA filters changing the room air 12 times in an hour should be used (Table 2) [98]. 

## In What Conditions Are HEPA Filters Effective?

The minimum acceptable limits that can lead to the development of IFDs are debatable. It is required that HEPA filters should remove the respirable particles from the environment at least at the Good Manufacturing Practices Class D level (Table 3). It was demonstrated in a Spanish study that if the limit for the room air is 0.5 CFU/m3, or in other words if the presence of 1 fungus colony in 2 m3 of air is allowed, it can lead to infections in high-risk patients [97].

## Evaluations of the Outcomes of HEPA Filter Use

The results of published studies on HEPA filters are summarized as follows [98,99,100,101,102,103,104,105]:

a. HEPA filters are effective to reduce the fungal load in the room air during and after construction.

b. HEPA filters were found to provide a more effective protection against invasive aspergillosis than amphotericin B during and after construction. 

c. Acceleration of the laminar flow increases the efficacy of HEPA filters. Fungal concentration in the air is correlated with a decrease in IFD incidence. 

d. Moreover, HEPA filters were shown to improve the general quality of life after transplantation.

## The Efficacy of Portable HEPA Filters

The CDC recommends the use of portable HEPA filters with rates of 300-800 cubic feet per minute to improve the removal process of respirable particles (Category 2). HEPA filters may be placed at different locations in and out of the room during and after construction until the surfaces are completely cleaned (Category 2). Microbiological analysis of the air samples during and immediately after construction is not recommended [98,99,100]. 

## Problematic Issues

Issues like patient-related comorbid conditions, the degree of immunosuppression, AFP, and microbiological quality of the water make it difficult to make scientific inferences. It has been reported that the gravity air-setting plate method is an applicable method in aerobiological monitoring of fungal spores. Petri plates involving Sabouraud agar media are placed in different areas of the rooms for 1 h with their lids open and each test is repeated 3 times. The samples are kept at 37 °C for 7 days. 

## Quality Control

The necessary protective precautions during transfer of the patients, primarily the hand-washing of the health care staff, and the necessary arrangements in terms of hygiene, waste control, and biosafety increase the efficacy of environmental control. 

## Antifungal Vaccines

### Dr. Zahit Bolaman

Aggressive chemotherapy or the use of agents leading to lymphocyte dysfunction such as rituximab and Campath and GVHD-related immunosuppression negatively affect the previous immunity achieved by vaccines in hematological malignant diseases [106]. As the risk for pneumococcal infection increases, patients are recommended to receive pneumococcal conjugate (PCV13) vaccine or pneumococcal polysaccharide vaccine (PPV23) before or during chemotherapy [107]. For inactivated influenza, hepatitis A, hepatitis B, meningococcus, conjugate haemophilus influenza, diphtheria-tetanus-pertussis, human papilloma, and poliovirus vaccinations, the country’s vaccination program is taken into account. It is not recommended that acute leukemia patients under induction, consolidation, or maintenance treatment or those receiving rituximab or alemtuzumab be vaccinated with any vaccine other than pneumococcal vaccine. Live vaccines including measles-mumps-scarlet fever, shingles, chicken pox, and polio are contraindicated before or during chemotherapy [108].

**Figure 1:**
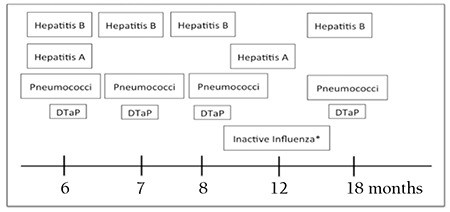
Vaccination program after allogeneic stem cell transplantation (modified from the Report from the International Consensus Conference on Clinical Practice in Chronic Graft-versus-host disease) [110]. DTaP: Diphtheria-tetanus-attenuated pertussis vaccine *Influenza vaccine is repeated each year.

The immune system is reorganized after allogeneic stem cell transplantation. Vaccination with PCV13 or PPV23, inactivated influenza, hepatitis A and B, conjugate haemophilus influenza B, diphtheria-tetanus-pertussis, conjugate meningococcus, or inactivated polio is recommended to be performed at 3-12 months after transplantation. General rules apply for the human papilloma virus vaccine. Live measles-mumps-scarlet fever vaccine is applied if the patient is seronegative, does not have GVHD, and is not receiving immunosuppressive treatment. Attenuated live influenza, live measles, mumps and measles-varicella, BCG, live shingles, varicella, and shingles vaccines are contraindicated (Table 4, Figure 1). Data are insufficient for typhoid fever and cholera vaccines [60,108,109,110].

The aggressive therapies used in hematological malignancies cause tissue destruction and immunosuppression, sometimes resulting in death due to fungal infections. Although some patients benefit from antifungal prophylaxis, the results are not very satisfying and optimal antifungal treatment strategies can only rescue 50% of patients. This is also associated with high economic cost. As a result, novel approaches are needed. Antifungal vaccines are developed for this purpose and show their effect by stimulating humoral or cellular immunity and by dendritic cells. For the full-blown effect of an antifungal vaccine, it is important to develop them against common fungal antigens (universal vaccines) [111]. The targeted antifungal determinants and their mechanisms are shown in Table 4. Successful results have been achieved in animal studies on fungal cell determinants, mainly laminarin, cell surface antigen, or dendritic cell-mediated vaccination [112]. Experimental animal studies are on-going with vaccines developed against Aspergillus, Candida, Cryptococcus, and pneumocystis infections, and 3 studies are being carried out in humans on recombinant NDV-3. Although lack of standardization, reduced immunogenicity, and difficulties in the vaccination of immunosuppressive individuals reduce the development speed of fungal vaccines, initial studies are promising in decreasing deaths related to fungal infections by fungal vaccinations in the future [112,113,114].

## Interpretation and Problematic Areas

### Dr. İhsan Karadoğan

Although prophylaxis in IFDs has become more evidence-based in recent years, there are still several gray areas and unresolved issues. A summary of these issues is presented in Table 5.

## Figures and Tables

**Table 1:**
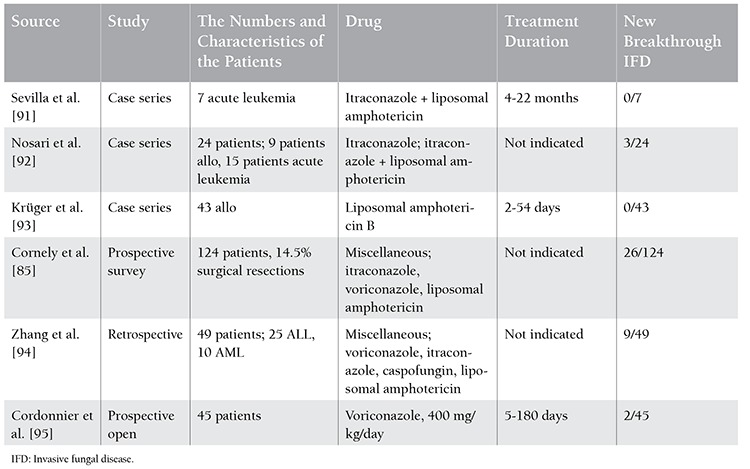
Studies on secondary prophylaxis for invasive fungal diseases (IFDs).

**Table 2:**
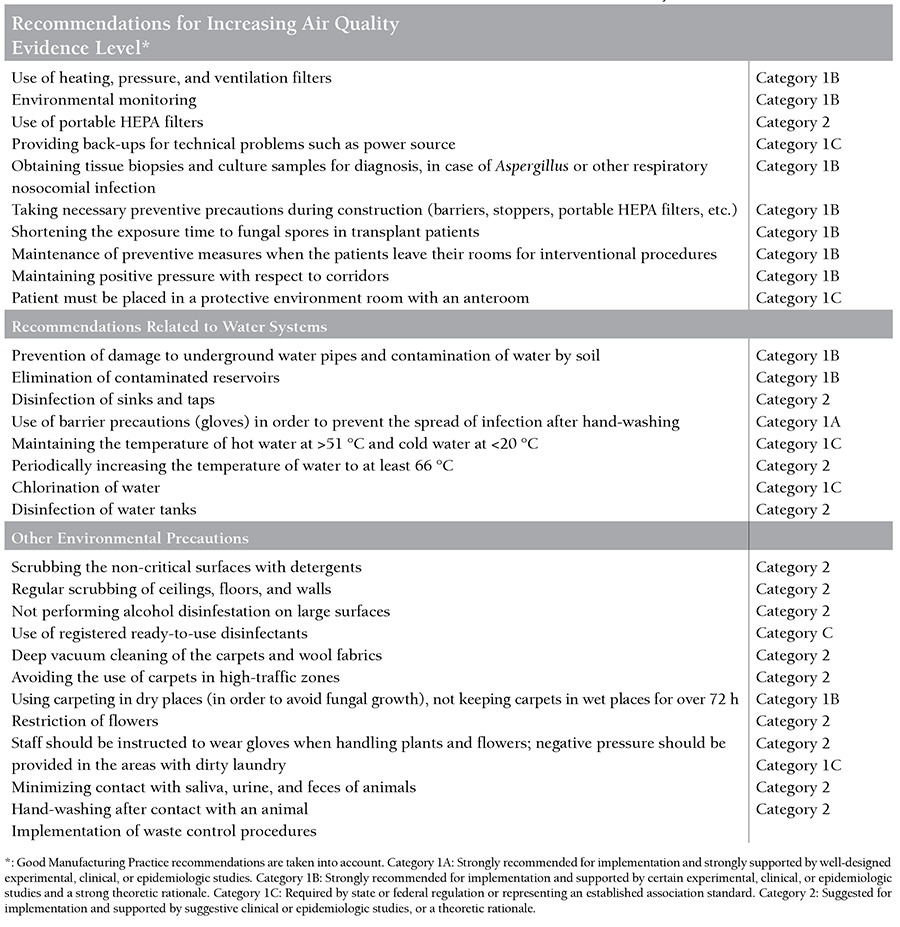
Recommendations of the CDC and the Healthcare Infection Control Practices Advisory Committee (HICPAC) [100].

**Table 3:**
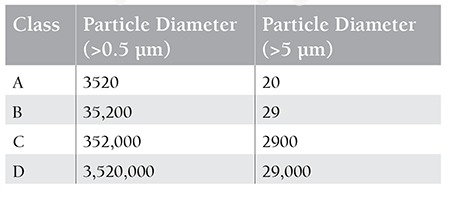
Clean room classification arranged according to the number of particles considering the particle size [100].

**Table 4:**
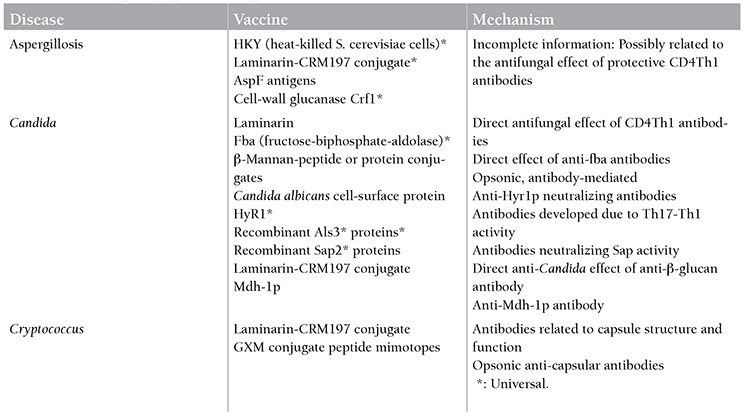
Immunological properties of fungal vaccines.

**Table 5:**
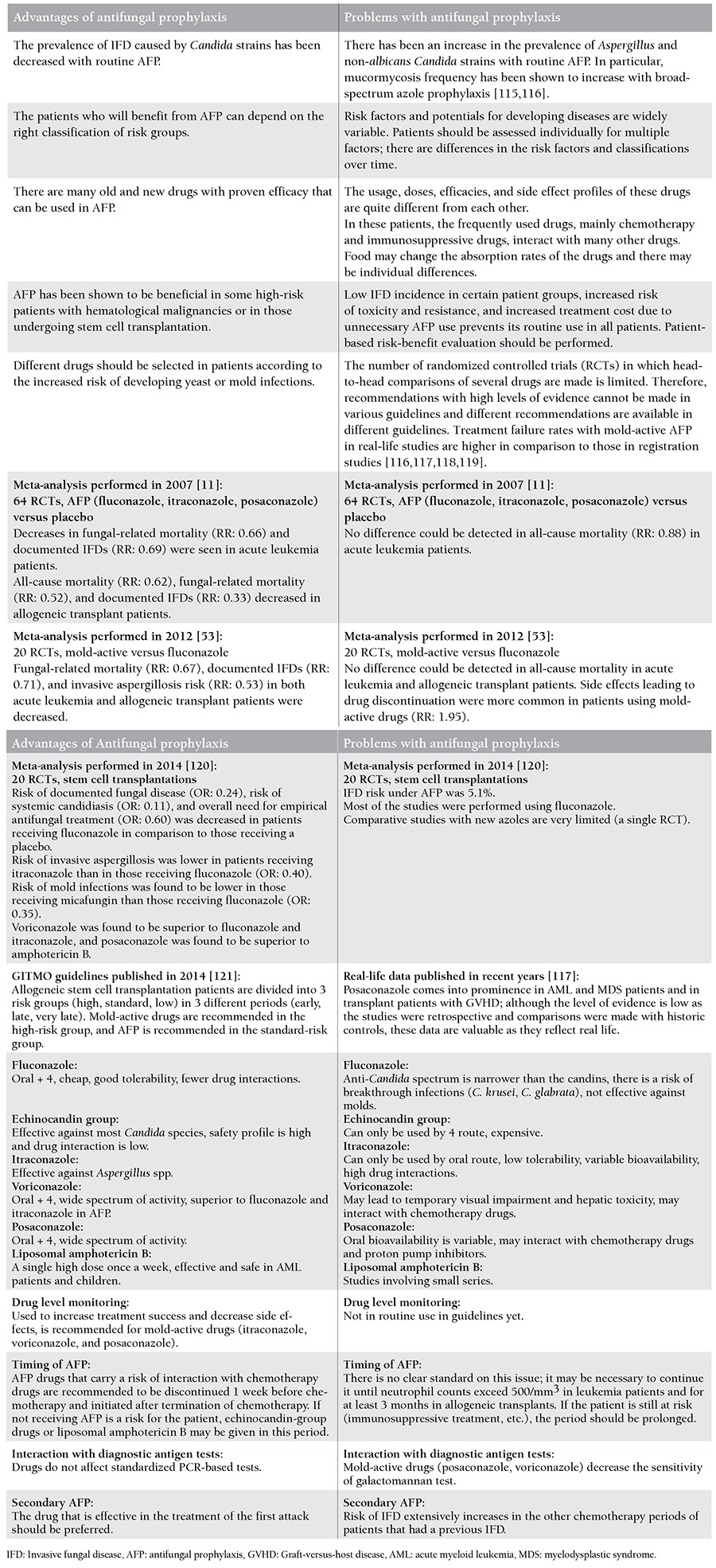
Pros and cons of antifungal prophylaxis.
